# Intravenous fish oil lipid emulsions in critically ill patients: an updated systematic review and meta-analysis

**DOI:** 10.1186/s13054-015-0888-7

**Published:** 2015-04-16

**Authors:** William Manzanares, Pascal L Langlois, Rupinder Dhaliwal, Margot Lemieux, Daren K Heyland

**Affiliations:** Intensive Care Unit, Faculty of Medicine-Universidad de la República (UdeLaR) University Hospital: Dr. Manuel Quintela, Italia Av. 14th Floor, Montevideo, 11600 Uruguay; Département de Anesthésie et de Réanimation, Faculté de Médecine et des Sciences de la Santé, Université de Sherbrooke, Centre Hospitalier Universitaire de Sherbrooke–Hôpital Fleurimont, Sherbrooke, PQ Canada; Clinical Evaluation Research Unit, Kingston General Hospital, Kingston, ON Canada; Department of Medicine, Queen’s University, Kingston, ON Canada

## Abstract

**Introduction:**

Intravenous fish oil (FO) lipid emulsions (LEs) are rich in ω-3 polyunsaturated fatty acids, which exhibit anti-inflammatory and immunomodulatory effects. We previously demonstrated that FO-containing LEs may be able to decrease mortality and ventilation days in patients who are critically ill. Since 2014, several additional randomized controlled trials (RCTs) of FO-containing LEs have been published. Therefore, the purpose of this systematic review was to update our previous systematic review with the aim of elucidating the efficacy of FO-containing LEs on clinical outcomes of patients who are critically ill.

**Methods:**

We searched electronic databases from 1980 to 2014. We included four new RCTs conducted in critically ill adult patients in which researchers evaluated FO-containing LEs in parenterally or enterally fed patients.

**Results:**

A total of 10 RCTs (n = 733) met inclusion criteria. The mean methodological score was 8 (range, 3 to 12). No effect on overall mortality was found. When we aggregated the results of five RCTs in which infections were reported, we found that FO-containing LEs significantly reduced infections (risk ratio (RR) = 0.64; 95% confidence interval (CI), 0.44 to 0.92; *P* = 0.02; heterogeneity *I*^2^ = 0%). Subgroup analysis demonstrated that predominantly enteral nutrition–based trials showed a tendency toward a reduction in mortality (RR = 0.69; 95% CI, 0.40 to 1.18; *P =*0.18; heterogeneity *I*^2^ =35%). High-quality trials showed a significant reduction in hospital length of stay (LOS) (weighted mean difference = −7.42; 95% CI, −11.89 to −2.94; *P* = 0.001), whereas low-quality trials had no effect (*P* = 0.45). The results of the test for subgroup differences in hospital LOS was significant (*P* = 0.001).

**Conclusion:**

FO-containing LEs may be associated with a reduction in infections and also could be associated with a reduction in duration of ventilation and hospital LOS. Further large-scale RCTs are warranted and should be aimed at consolidating potential positive treatment effects.

## Introduction

Lipid emulsions (LEs), which are an integral part of a parenteral nutrition (PN) regimen, provide exogenous fatty acids, which are commonly used by cells as an energy-dense source of fuel calories (approximately 9 kcal/g), cell membrane components and biologically active substrates [1-3]. In patients who are critically ill, commonly used LEs have provided long-chain triglycerides (LCTs), particularly soybean oil (SO), with a high percentage of ω-6 polyunsaturated fatty acids (PUFAs; 18:2 ω-6) [4]. Nonetheless, over the last few decades, several different SO-sparing strategies have been developed, which are defined as alternative oil-based LEs. Fish oil (FO)-containing LEs are a type of alternative LE that are high in ω-3 PUFAs (18:3 ω-3). Examples are eicosapentaenoic acid (EPA) and docosahexaenoic acid (DHA) [5]. According to the capacity of EPA and DHA to modulate the synthesis of eicosanoids, the activity of nuclear receptor and nuclear transcription factors, and the production of resolvins, these fatty acids have long been recognized as having anti-inflammatory and immunomodulatory effects [6]. In a secondary analysis of data from the International Nutrition Survey, Edmunds *et al*. [7] demonstrated that FO-containing LEs were able to reduce the duration of mechanical ventilation (MV) and time of intensive care unit (ICU) discharge alive compared with SO-based LEs. Over the last few years, there have been several RCTs on FO-containing LEs in patients who are critically ill in which investigators reported relevant clinical outcomes, which have been included in previous meta-analyses [8-10].

In 2012, in a systematic review and meta-analysis, Pradelli *et al*. [8] concluded that in elective surgery and critically ill patients, parenteral FO-containing LEs were associated with a significant reduction in infections, although Palmer *et al*. [9], in another systematic review, were unable to find similar results in ICU patients.

In 2013, after aggregating six RCTs evaluating the effect of parenteral FO-containing LEs on relevant clinical outcomes in a heterogeneous ICU patient population [10], we demonstrated that FO-containing LEs may be able to decrease mortality (risk ratio (RR), 0.71; 95% confidence interval (CI), 0.49 to 1.04; *P* = 0.08) and also reduce the duration of MV (weighted mean difference (WMD) = −1.41; 95% CI, −3.43 to 0.61; *P* = 0.17) [10]. Nonetheless, since 2014, several RCTs evaluating clinical effects of FO-containing LEs as compared with other LEs (SO, medium-chain triglycerides (MCTs) and/or LCTs or olive oil (OO)) in ICU patients being treated with PN- and EN-based strategies have been published. Therefore, with the aim of elucidating the efficacy of parenteral FO-containing LEs in patients who are critically ill, we performed an update of our previous systematic review and meta-analysis of the literature.

## Methods

### Search strategy and study identification

A literature review was conducted to identify in MEDLINE, Embase, CINAHL, the Cochrane Central Register of Controlled Trials and the Cochrane Database of Systematic Reviews all relevant RCTs published between 1980 and November 2014. The following keywords or medical subject headings were used: “randomized,” “clinical trial,” “nutrition support,” “artificial feeding,” “parenteral nutrition,” “pharmaconutrition,” “omega-3 fatty acids,” “fish oils,” “lipid emulsions,” “intensive care,” “critical illness” and “critically ill.” We did not restrict our search to only articles written in English.

### Eligibility criteria

Trials were included if they met the following characteristics:

1. *Type of study*: RCT with a parallel group.
2. *Population*: critically ill adult patients (≥18 years of age), defined as patients admitted to an ICU. If the study population was unclear, we considered a mortality rate higher than 5% in the control group to be consistent with critical illness. We excluded RCTs performed in elective surgery patients (such as open-heart surgery patients) even if patients were cared for in an ICU in the postoperative period.
3. *Intervention*: intravenous FO-containing LEs as part of PN or as a pharmaconutrient strategy (enterally fed patients).
4. *Control*: EN, PN with SO-based LEs or a non-FO-based LE, as well as saline solution. Non-FO LEs and saline solution were defined as non-FO lipids or non-FO strategies.
5. *Outcomes*: Overall mortality was the primary outcome for this meta-analysis. Secondary outcomes were infections, ICU and hospital length of stay (LOS) and MV days. As in previous meta-analyses conducted by our group, we excluded those trials that reported only nutrition, biochemical, metabolic or immunologic outcomes. The methodological quality of the included trials was assessed in duplicate independently by two reviewers using a data abstraction form with a scoring system [11] from 0 to 14 according to the following criteria:
  - The extent to which randomization was concealed
  - Blinding
  - Analysis based on the intention-to-treat (ITT) principle
  - Comparability of groups at baseline
  - Extent of follow-up
  - Description of treatment protocol
  - Cointerventions
  - Definition of clinical outcomes

Consensus between both reviewers on the individual scores of each of the categories was obtained. We attempted to contact the authors of included studies and requested additional information not contained in published articles. We designated studies as level I if all of the following criteria were fulfilled: concealed randomization, blinded outcome adjudication and an ITT analysis, which are the strongest methodological tools to reduce bias. A study was considered as level II if any one of the above-described characteristics were unfulfilled.

### Data synthesis

The primary outcome of the systematic review was mortality (hospital mortality or if not reported, 28 days or ICU mortality). The definitions of infectious complications as defined by authors of individual trials were used. We analyzed data using RevMan 5.3 (Cochrane IMS, Oxford, UK) with a random effects model. We combined data from all trials to estimate the pooled risk ratio (RR) with 95% confidence intervals (CIs) for mortality and infections and overall weighted mean difference (WMD) with 95% confidence intervals for LOS data. Pooled RRs were calculated using the Mantel-Haenszel estimator, and WMDs were estimated by the inverse variance approach. The random effects model of DerSimonian and Laird was used to estimate variances for the Mantel-Haenszel [12] and inverse variance estimators. RRs were undefined and excluded for studies with no event in either arm. Heterogeneity was tested by a weighted Mantel-Haenszel χ^2^ test and quantified by the I^2^ statistic as implemented in RevMan. Differences between subgroups were analyzed using the test of subgroup differences described by Deeks *et al*. [13], and the results expressed using the *P*-values.

The possibility of publication bias was assessed by generating funnel plots and testing asymmetry of outcomes using methods proposed by Rucker et al. [14]. We considered *P* <0.05 to be statistically significant and *P* <0.20 as an indicator of trend.

### Subgroup analysis

We performed a predefined subgroup analysis to assess a number of possible influences on the effects of intravenous FO-containing LEs on clinical outcomes. We first examined the effect of parenteral FO-containing LEs in the context of PN (PN-based trials) versus predominantly EN-based trials [15-18], which included EN alone, EN with PN, EN with oral (PO) or EN, PN and PO together. In addition, as the trial quality can influence clinical findings, we postulated that trials with lower quality (defined as level II studies) may demonstrate a greater treatment effect than those trials with higher quality (level I studies), which were previously defined.

## Results

**Figure 1:**
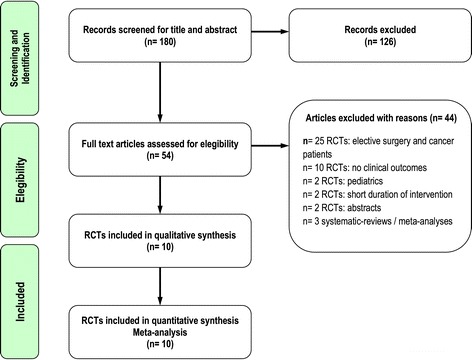
Flow diagram of the literature search according to the Quality of Reporting of Meta-analyses statement [72].

A total of 54 relevant citations were identified in the search of electronic bibliographic databases and a review of reference lists in related articles. We excluded 44 trials for the following reasons: 25 trials [19-43] did not include ICU patients (mostly elective surgery and cancer patients); 10 trials [44-53] did not evaluate clinically important outcomes; 2 trials [54,55] were published as abstracts, and we were unable to obtain the data from the authors to complete our data abstraction process; 2 trials [56,57] were conducted in a pediatric population; 2 trials [58,59] had a short duration of intervention (a single infusion during the study period); and 3 articles [8,9,60] were systematic reviews and meta-analyses (Figure 1).

In the end, ten RCTs [15-18,61-66], including four new trials [17,18,65,66] published since our last meta-analysis, met the inclusion criteria and were finally included in this systematic review (see Tables 1 and 2). The ten trials comprised an aggregate total of 733 patients. There was considerable heterogeneity in the interventions tested in these trials. Among RCTs in PN-fed patients, three trials [63-65] compared a LCT + MCT + FO LE with an MCT + LCT LE, two trials [61,62] compared a FO + LCT LE with a LCT LE and one trial [66] compared a FO + OO LE with an OO-containing LE. Among the four RCTs in which investigators evaluated predominantly EN-fed patients [15-18], one trial [16] compared supplementation of EN or PO diet with a parenteral FO-containing LE to EN or PO diet with intravenous normal saline, one trial [15] compared supplementation of EN with a parenteral FO-containing LE to EN alone, one trial [17] compared supplementation of EN, PN or PO diet with a parenteral FO-containing LE to EN or PN or PO diet alone, and one trial [18] compared supplementation of EN or PN with a parenteral FO-containing emulsion to EN or PN alone. We reached 100% agreement for inclusion of trials in the present review. The mean methodological score of all trials was 9 (range, 3 to 12). Randomization was concealed in six (60%) of the ten trials, ITT analysis was performed in eight trials (80%) and seven trials (70%) were double-blinded. There were five level I studies [15,61-64] and five level II studies [16-18,61,66]. The details of the methodological quality of the individual trials are shown in Table 1.

**Table 1:** **Randomized clinical trials evaluating parenteral fish oil–containing lipid emulsions in critically ill patients** ^**a**^

**Study**	**Population**	**Methods (score)**	**Intervention**	**Mortality (%)** ^**b**^		**Infections (%)** ^**c**^	
**FO**							
**Grecu** ***et al*** **., 2003** ^**d**^ [62]	Patients with abdominal sepsis	C.Random: Yes	PN + Omegaven (10% FO; Fresenius Kabi, Bad Homburg, Germany) plus LCT vs. PN with LCT	**Omegaven + LCT**	**LCT**	**Omegaven**	**LCT**
	N =54	ITT: Yes		**ICU**	**ICU**	**VAP**	**VAP**
	(15 of 54 in ICU)	Blinding: Double		2/28 (7)	3/26 (12)	0/8	1/7 (14)
		(12)					
**Friesecke** ***et al*** **., 2008** [63]	Medical ICU patients	C.Random: Yes	PN + Lipofundin (B Braun, Melsungen, Germany) MCT (50% LCT + 50% MCT) + Omegaven (10% FO) vs. PN with Lipofundin MCT (50% LCT + 50% MCT)	**MCT + LCT + FO**	**LCT + MCT**	**MCT + LCT + FO**	**LCT + MCT**
	N =166	ITT: Yes		**28 days**	**28 days**	10/83 (12)	11/82 (13)
		Blinding: Double		18/83 (22)	22/82 (27)		
		(10)					
**Wang** ***et al*** **., 2009** [61]	Severe acute pancreatitis patients in ICU	C.Random: No	PN + Omegaven (10% FO) plus Lipovenos (LCT, soybean oil; Fresenius Kabi) (ω3:ω6 ratio was 1:4) vs. PN with Lipovenos (LCT, soybean oil). Both received same amounts of lipids (1 g/kg/day)	**Omegaven**	**LCT**	**Omegaven**	**LCT**
	N =56	ITT: Yes		**ICU**	**ICU**	6/28 (21)	9/28 (32)
		Blinding: Double		0/28 (0)	2/28 (7)		
		(11)					
**Barbosa** ***et al*** **., 2010** [64]	ICU patients with SIRS or sepsis requiring PN	C.Random: Yes	PN + Lipolus (50% MCT, 40% LCT soybean oil, 10% FO; B Braun) vs. PN with NuTRIflex Lipid Special (50% MCT, 50% LCT, soybean oil; B Braun). Both received same amounts of lipids (about 1 g/kg/day)	**MCT + LCT + FO**	**MCT + LCT**	**MCT + LCT + FO**	**MCT + LCT**
	N =25	ITT: Yes		**5 days**	**5 days**	NR	NR
		Blinding: Single		2/13 (15)	1/10 (10)		
		(10)		**28 days**	**28 days**		
				4/13 (31)	4/10 (40)		
**Gupta** ***et al*** **., 2011** [15]	ICU patients with suspected ARDS	C.Random: Yes	EN (standard diet) + Omegaven 10% (ω3:ω6 ratio was 1:4) vs. EN (standard diet)	**Omegaven**	**Standard**	**NR**	**NR**
	N =61	ITT: Yes		**ICU**	**EN**		
		Blinding: Double		7/31 (23)	**ICU**		
		(9)		**Hospital**	13/30 (43)		
				9/31 (29)	**Hospital**		
					14/30 (47)		
**Khor** ***et al*** **., 2011** [16]	ICU patients with severe sepsis/septic shock	C.Random: Yes	Supplementation with 100 ml of 10% Omegaven (10 g of refined FO, EPA 12.5 to 28.2 g/L, DHA 14.4 to 30.9 g/L) vs. 100 ml of 0.9% normal saline	**NR**	**NR**	**NR**	**NR**
	N =28	ITT: No					
		Blinding: Double					
		(8)					
**Burkhart** ***et al*** **., 2013** [18]	ICU patients with sepsis	C.Random: ?	2 ml/kg/day Omegaven vs. no parenteral FO. Both groups received EN and/or PN without added FO at the discretion of the clinician.	**Omegaven**	**No Omegaven**	**NR**	**NR**
	N =50	ITT: Yes		**Hospital**	**Hospital**		
		Blinding: Single (assessor)		13/25 (52)	13/25 (52)		
		(8)					
**Grau-Carmona** ***et al*** **., 2014** [65]	Medical and surgical patients requiring PN	C.Random: Yes	PN + Lipoplus (50% MCT, 40% LCT soybean oil, 10% FO; B Braun) vs. PN + Lipofundin (50% LCT + 50% MCT)	**MCT + LCT + FO**	**MCT + LCT ICU**	**MCT + LCT + FO**	**MCT + LCT**
	N =175	ITT: Yes		**ICU**	16/78 (20.5)	17/81 (21)	29/78 (37.2)
		Blinding: Double		26/81 (32.5)	**Hospital**		
		(10)		**Hospital**	6/78 (9.7)		
				6/81 (11.1)	**6 months**		
				**6 months**	2/78 (3.6)		
				2/81 (4.3)			
**Gultekin** ***et al*** **., 2014** [66]	ICU patients needing TPN	C.Random: ?	PN + Omegaven (10% FO) plus ClinOleic (80% olive oil, 20% soybean oil; Baxter Healthcare, Compton, UK) vs. PN + ClinOleic	**Omegaven + olive oil**	**Olive oil**	**NR**	**NR**
	N =58	ITT: Other		**Unspecified**	**Unspecified**		
		Blinding: Double		8/16 (50)	7/16 (44)		
		(3)					
**Hall** ***et al*** **., 2014** [17]	ICU patients with sepsis	C.Random: ?	Omegaven dosed at 0.2 g of FO/kg/day given at a rate of 0.05 g of FO/kg/day vs. no FO. Both groups received EN and/or PN at the discretion of the clinician.	**Omegaven**	**No Omegaven**	**Omegaven**	**No Omegaven**
	N =60	ITT: Yes		**Hospital**	**Hospital**	3/30 (10)	5/30 (16.7)
		Blinding: No		4/30 (13.3)	9/30 (30)		
		(9)		**28 days**	**28 days**		
				4/30 (13.3)	8/30 (26.7)		

^a^These studies compared fish oil (ω-3)–containing emulsions in parenteral nutrition–fed patients with long-chain triglycerides or long-chain triglycerides **+** medium-chain triglycerides. ARDS, Acute respiratory distress syndrome; C.Random, Concealed randomization; DHA, Docosahexaenoic acid; EPA, Eicosapentaenoic acid; FO, Fish oil; ICU, Intensive care unit; ITT, Intention to treat; LCT, Long-chain triglycerides; LE, Lipid emulsion; MCT, Medium-chain triglycerides; NA, Non-attributable; NR, Non-reported; PN, Parenteral nutrition; SIRS, Systemic inflammatory response syndrome; TPN, Total parenteral nutrition; VAP, Ventilator-associated pneumonia; ?, Doubtful. ^b^Hospital mortality unless specified. ^c^Number of patients with infections unless specified. ^d^Data obtained from author, 8 out of 28 in Omegaven (Fresenius Kabi, Bad Homburg, Germany) and 7 of 26 in LCT group were in ICU.

**Table 2:** **Outcomes of included trials on fish oil strategies using lipid emulsions** ^**a**^

**Study**	**LOS in days**		**Ventilator days**		**Other**	
**FO**						
**Grecu** ***et al*** **., 2003** ^**b**^ [62]	**Omegaven** (Fresenius Kabi, Bad Homburg, Germany)	**LCT**	**Omegaven**	**LCT**	**Omegaven**	**LCT**
	**ICU**	**ICU**	2.83 ± 1.62 (8)	5.23 ± 2.80 (7)	**Patients undergoing reoperation for septic episode**	
	3.32 ± 1.48 (8)	9.28 ± 3.08 (7)			2/28 (7)	8/26 (31)
	**Hospital**	**Hospital**				
	11.68 ± 2.04 (28)	20.46 ± 3.27 (26)				
**Friesecke** ***et al*** **., 2008** [63]	**FO**	**LCT**	**LCT + MCT + FO**	**LCT + MCT**	**LCT + MCT + FO**	**LCT + MCT**
	**ICU**	**ICU**	22.8 ± 22.9 (83)	20.5 ± 19.0 (82)	**Urinary tract infections**	
	28 ± 25 (83)	23 ± 20 (82)			6/83 (7)	4/82 (5)
					**Catheter-related infections**	
					1/83 (1)	3/83 (4)
					**Total EN energy intake (kcal/kg)**	
					22.2 ± 5.5	21.6 ± 5.6
**Wang** ***et al*** **., 2009** [61]	NR	NR	NR	NR	**Omegaven**	**LCT**
					**Surgery of infected pancreatic necrosis**	
					3/28 (11)	6/28 (21)
**Barbosa** ***et al*** **., 2010** [64]	**MCT + LCT + FO**	**MCT + LCT**	**MCT + LCT + FO**	**MCT + LCT**	**MCT + LCT+ FO**	**MCT + LCT**
	**ICU**	**ICU**	10 ± 14.4 (13)	11 ± 12.64 (10)	2,057 ± 418 kcal	1,857 ± 255 kcal
	12 ± 14.4^c^ (13)	13 ± 12.6^c^ (10)				
	**Hospital**	**Hospital**				
	22 ± 25.2^c^ (13)	55 ± 50.6^c^ (10)				
**Gupta** ***et al*** **., 2011** [15]	**Omegaven**	**Standard EN**	**Omegaven**	**Standard EN**		
	**ICU**	**ICU**	11.78 ± 10.63 (31)	10.71 ± 14.55 (30)		
	15.96 ± 7.57 (31)	15.88 ± 6.47 (30)				
	**Hospital**	**Hospital**				
	21.5 ± 13.49 (31)	26.63 ± 18.22 (30)				
**Khor** ***et al*** **., 2011** [16]	**Omegaven**	**Saline**	**Omegaven**	**Saline**		
	**ICU**	**ICU**	13.0 ± 10.1 (9)	11.6 ± 9.5 (5)		
	10.3 ± 8.4 (14)	8.4 ± 6.5 (13)				
	**Hospital**	**Hospital**				
	19.6 ± 7.4 (14)	17.5 ± 6.0 (13)				
**Burkhart** ***et al*** **., 2013** [18]	**Omegaven**	**No Omegaven**	**NR**	**NR**	**Omegaven**	**No Omegaven**
	**ICU**	**ICU**			**Subsyndromal delirium**	
	5 (3 to 22)	6 (2 to 33)			5 (25)	6 (29)
					**Sepsis associated delirium**	
					**15 (75)**	15 (71)
**Grau-Carmona** ***et al*** **., 2014** [65]	**MCT + LCT + FO**	**MCT + LCT**	**MCT + LCT + FO**	**MCT + LCT**	**MCT + LCT+ FO**	**MCT + LCT**
	**ICU**	**ICU**	8.41 ± 6.61	9.2 ± 6.0	**Parenteral lipid intake [(g/kg BW)/d]**	
	18.9 ± 15.5	21.8 ± 20.9			1.04 ± 0.12	1.05 ± 0.13
	**Hospital**	**Hospital**			**PN kcal**	
	41.1 ± 41.0	42.5 ± 28.5			1,737 ± 353	1,782 ± 312
**Gultekin** ***et al*** **., 2014** [66]	**Omegaven + olive**	**Olive oil**	**NR**	**NR**	**Omegaven + olive oil**	**Olive oil**
	**Hospital**	**Hospital**				
	31.6 ± 4.3	30.6 ± 4.3			27.5 ± 1.5 **kcal/kg/day**	15.8 ± 1.5 **kcal/kg/day**
					1.3 ± 0.2 **g protein/kg/day**	1.1 ± 0.1 **g protein/kg/day**
**Hall** ***et al*** **., 2014** [17]	**Omegaven**	**No Omegaven**	**NR** (reported as free ventilator days)	**NR** (reported as free ventilator days)		
	**ICU**	**ICU**				
	8.8 ± 7.7	12.3 ± 12.4				
	**Hospital**	**Hospital**				
	26.7 ± 18.2	33.5 ± 30.4				

^a^These studies compared fish oil (ω-3)–containing emulsions in parenteral nutrition–fed patients vs. LCT or LCT + MCT. EN, Enteral nutrition; FO, Fish oil; ICU, Intensive care unit; LCT, Long-chain triglycerides; MCT, Medium-chain triglycerides; ITT, Intention to treat; NR, Not reported. ^b^Data obtained from author, 8 of 28 in Omegaven (Fresenius Kabi, Bad Homburg, Germany) group and 7 of 26 in LCT group were in the ICU. ^c^Converted standard error of the mean to standard deviation.

### Meta-analyses of primary and secondary outcomes

#### Primary outcome

##### Mortality

**Figure 2:**
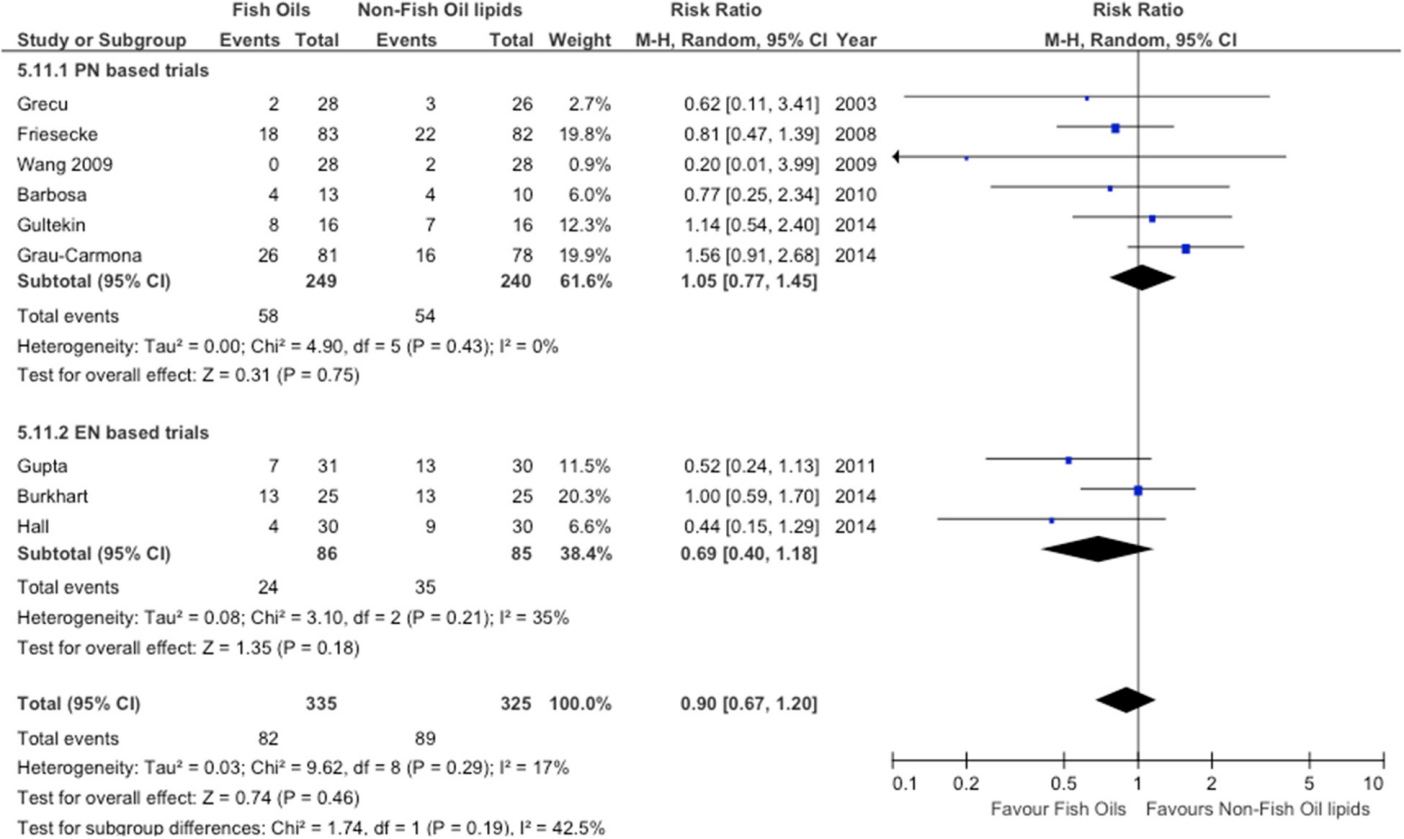
Effects on mortality of fish oil lipid emulsion strategies (n =9). CI, Confidence interval; EN, Enteral nutrition; LCT, Long-chain triglycerides; MCT, Medium-chain triglycerides; M-H, Mantel-Haenszel test; PN, Parenteral nutrition.

When the data from nine RCTs [15,17,18,61-66] evaluating mortality as one of the outcomes were aggregated, FO-containing strategies did not achieve a statistically significant reduction in mortality (RR = 0.90; 95% CI, 0.67 to 1.20; *P* = 0.46; heterogeneity *I*^2^ = 0%) (see Figure 2). Heterogeneity was not significant (*P* = 0.29).

#### Secondary outcomes

##### Infectious complications

**Figure 3:**
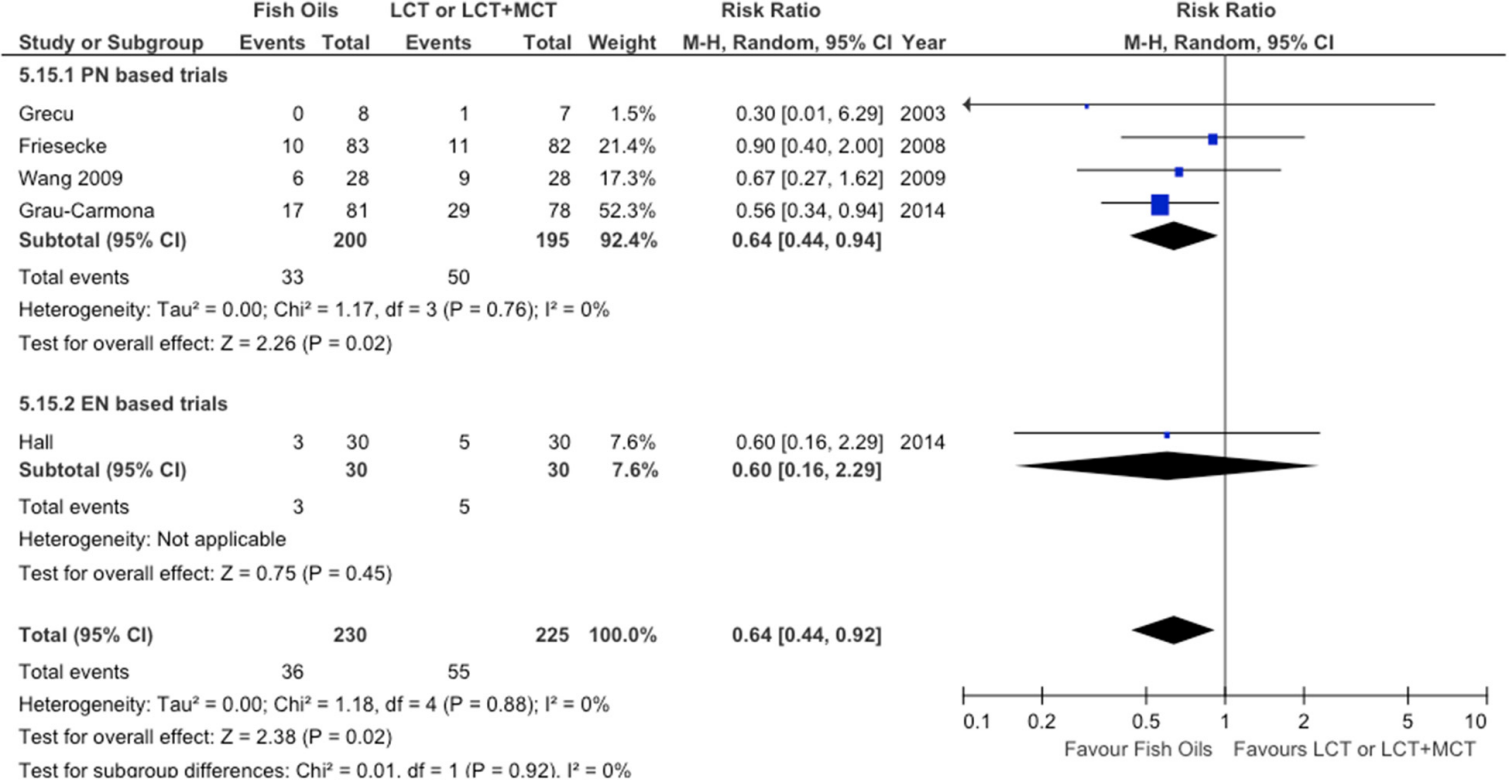
Effects on infections of parenteral fish oil containing emulsions (n =5). CI, Confidence interval; EN, Enteral nutrition; LCT, Long-chain triglycerides; MCT, Medium-chain triglycerides; M-H, Mantel-Haenszel test; PN, Parenteral nutrition.

When the results of five RCTs [17,61-63,65] that reported infectious complications were aggregated, FO-containing LEs significantly reduced infections (RR = 0.64; 95% CI, 0.44 to 0.92; *P* = 0.02; heterogeneity *I*^2^ = 0%) (see Figure 3). Similarly, after excluding the Grecu *et al*. [62] study because the authors reported only ventilator-associated pneumonia (VAP), the same effect was shown (RR = 0.65; 95% CI, 0.44 to 0.94; *P* = 0.02; heterogeneity *I*^2^ = 0%).

##### Mechanical ventilation

**Figure 4:**
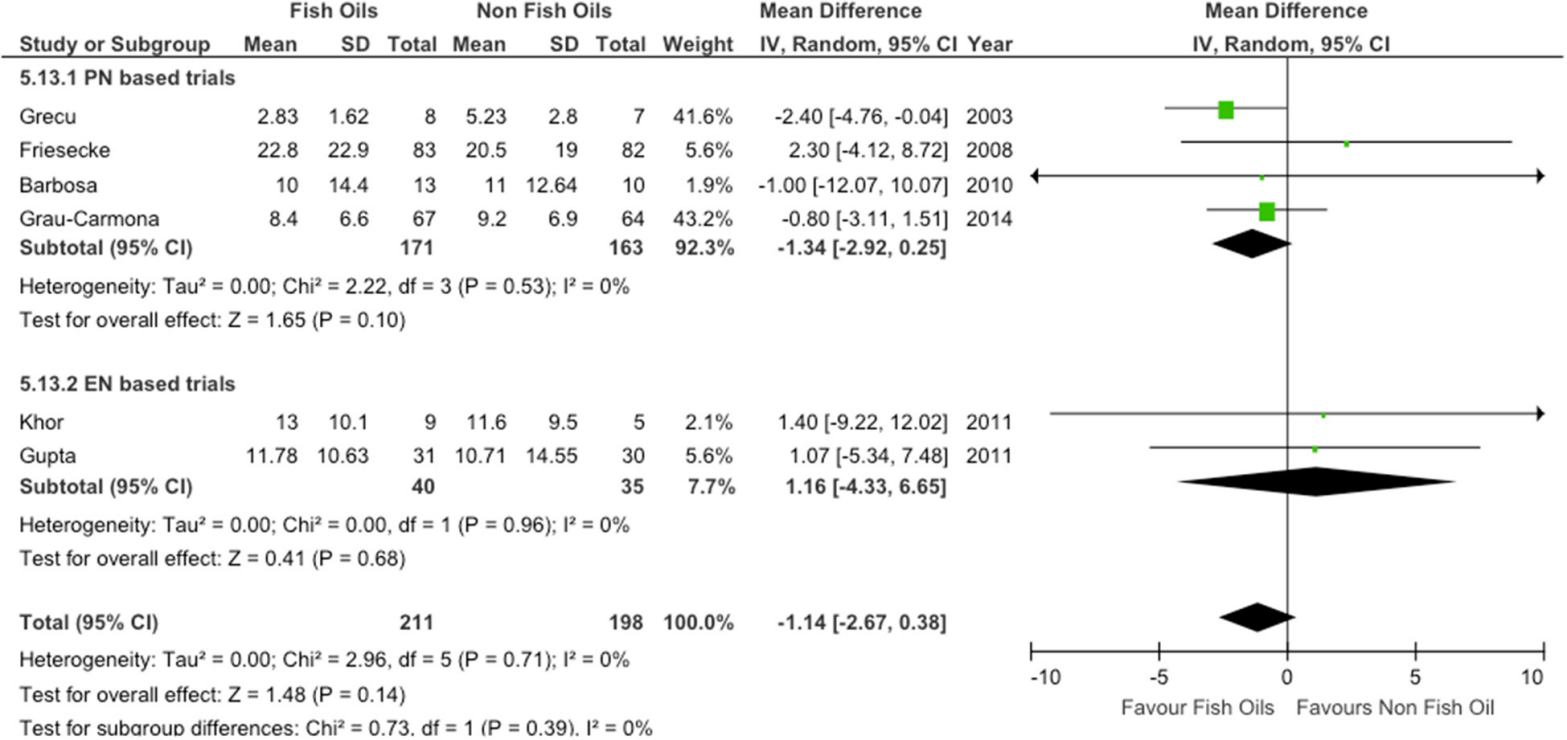
Effects on mechanical ventilation days of parenteral fish oil containing emulsions (n =6). CI, Confidence interval; EN, Enteral nutrition; IV, Inverse Variance; M-H, Mantel-Haenszel test; PN, Parenteral nutrition; SD, Standard deviation.

The aggregation of six RCTs [15,16,62-65] on FO-containing LEs in which the researchers reported on MV showed a trend toward reduction in the number of days on MV (WMD, −1.14; 95% CI, −2.67 to 0.38; *P* = 0.14; heterogeneity *I*^2^ = 0%) (see Figure 4).

##### Hospital length of stay

**Figure 5:**
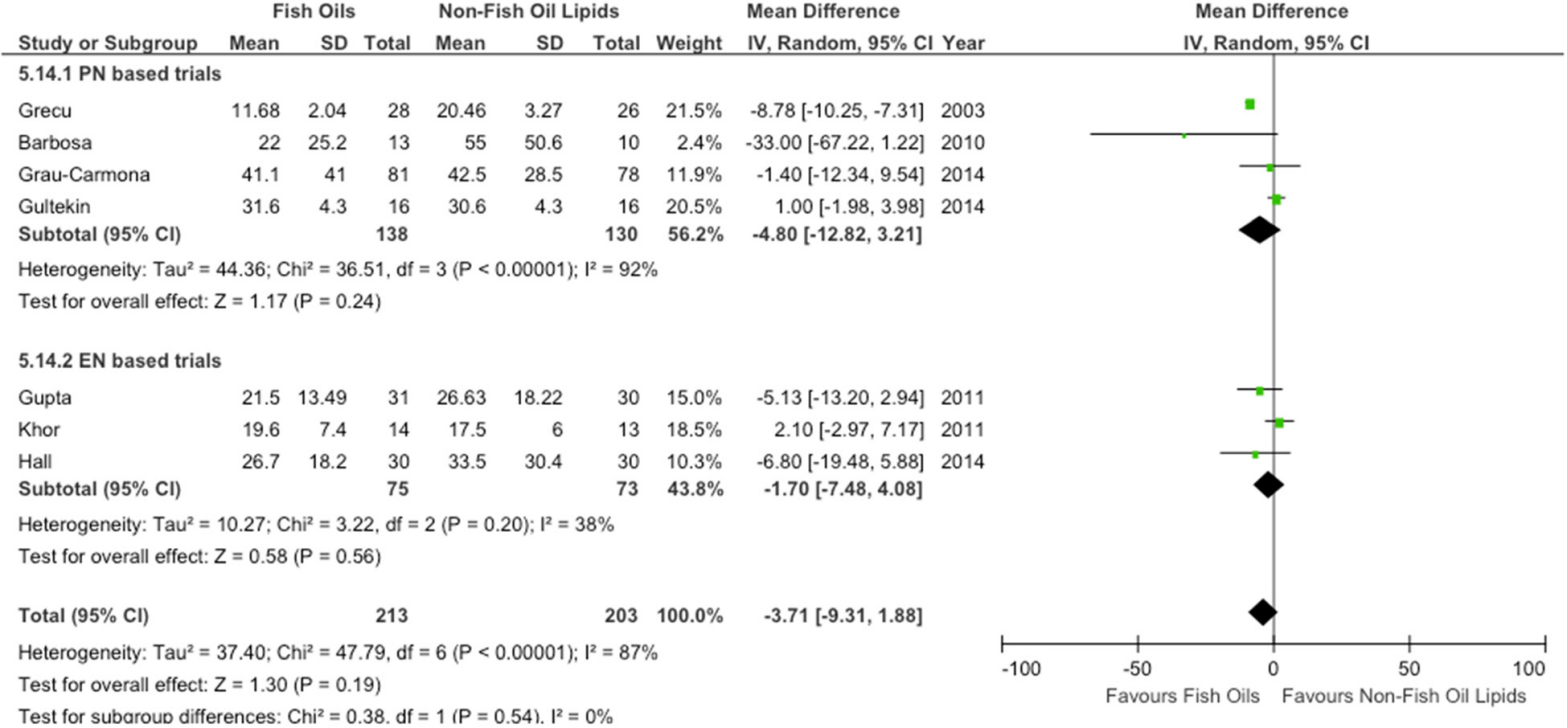
Effects on hospital length of stay of parenteral fish oil containing emulsions (n =7). CI, Confidence interval; EN, Enteral nutrition; IV, Inverse Variance; M-H, Mantel-Haenszel test; PN, Parenteral nutrition; SD, Standard deviation.

When the data from seven RCTs [15-17,62,64-66] in which researchers reported on hospital length of stay were aggregated, FO-containing strategies showed a trend toward reduction in hospital LOS, without reaching statistical significance (WMD = −3.71; 95% CI, −9.31 to 1.88; *P* = 0.19) (see Figure 5). The heterogeneity was significant (*P* <0.00001; *I*^*2*^ = 87%).

##### ICU length of stay

**Figure 6:**
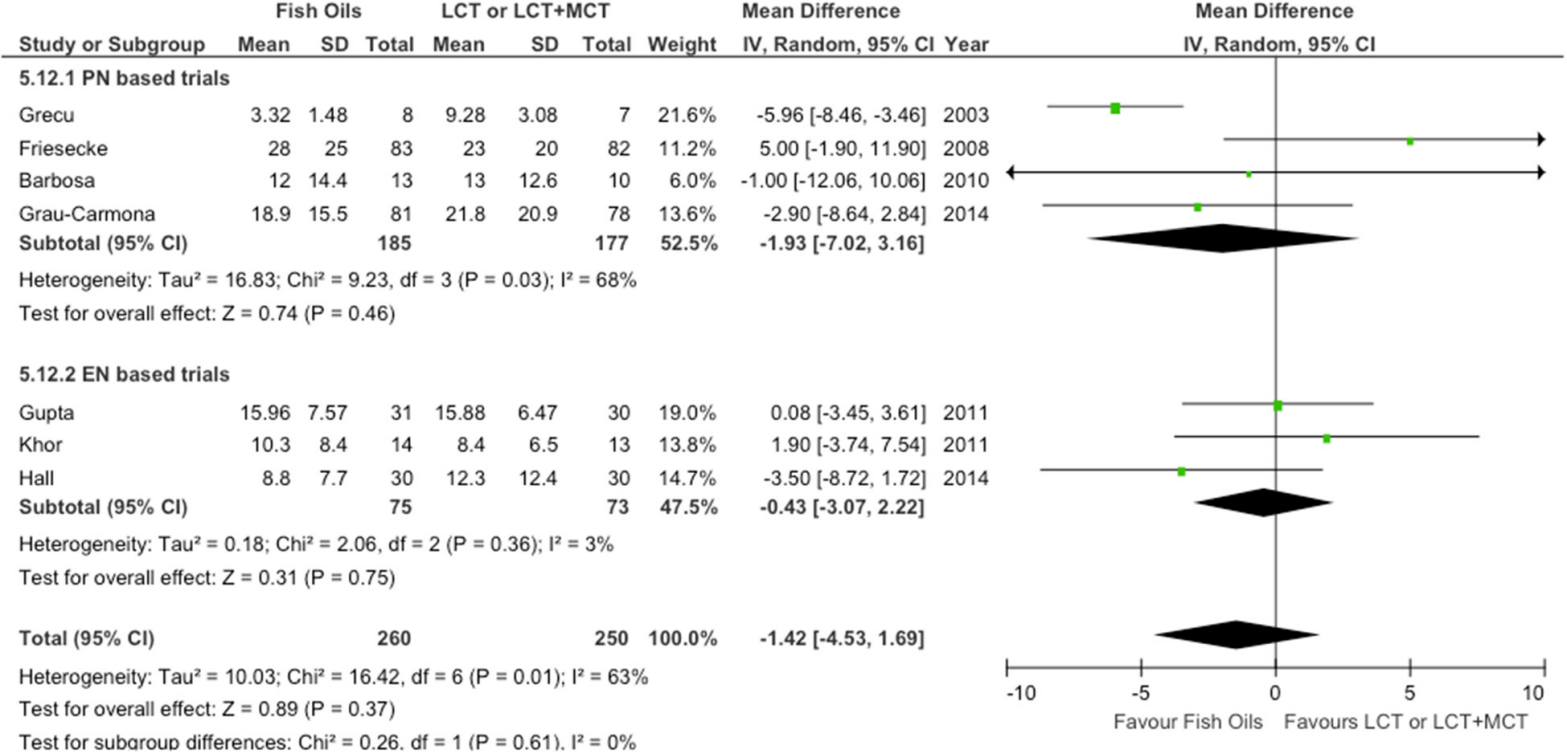
Effects on ICU length of stay of parenteral fish oil containing emulsions (n =7). CI, Confidence interval; EN, Enteral nutrition; IV, Inverse Variance; M-H, Mantel-Haenszel test; PN, Parenteral nutrition; SD, Standard deviation.

The seven RCTs [15-17,62-65] evaluating ICU length of stay were aggregated, but we did not find any effects related to FO-containing LEs in parenterally fed patients (WMD = −1.42; 95% CI, −4.53 to 1.69; *P* = 0.37) (see Figure 6). However, the heterogeneity analysis was statistically significant (*P* = 0.01; *I*^2^ = 63%).

### Subgroup analyses

#### Parenteral nutrition–based trials versus predominantly enteral nutrition–based trials

In six trials, researchers investigated the administration of FO-containing LEs in parenterally fed patients [61-66], whereas in four trials [15-18], parenteral FO supplementation was studied in the context of patients receiving predominantly EN. There were no significant differences in any endpoint between trials of PN- versus EN-based nutritional strategies. However, in those EN-based trials [15,17,18] in which patients received intravenous FO-containing LEs, this pharmaconutrient strategy showed a tendency toward a reduction in mortality (RR = 0.69; 95% CI, 0.40 to 1.18; *P* = 0.18; heterogeneity *I*^2^ = 35%) (see Figure 2). When we examined the effect of FO-containing LEs in PN-based trials versus predominantly EN-based trials, the test for subgroup differences on overall mortality showed a trend (*P* = 0.19).

#### Effect of study quality on outcomes

Similarly to low-quality trials [17,18,61,66], the higher-quality trials [15,62-65] did not show any effect on mortality (*P* = 0.58 in both subgroups). Nonetheless, a statistically significant effect of FO-containing LEs on the reduction of infections was shown in the higher-quality trials [62,63,65] (RR = 0.64; 95% CI, 0.42 to 0.97; *P* = 0.04; heterogeneity *I*^2^ = 0%), whereas trials with lower methods scores did not show any significant effect (RR = 0.65; 95% CI, 0.31 to 1.35; *P* = 0.25; heterogeneity *I*^2^ = 0%). However, the overall tests for significance did not reveal statistically significant differences between these subgroups (*P* = 0.97).

The effect of study quality on MV did not show any significant effect, although one low-quality trial [16] explored this outcome. The test for subgroup differences on MV days was not significant (*P* = 0.64). With regard to hospital LOS, higher-quality trials [15,62,64,65] showed a significant reduction (WMD = −7.42; 95% CI, −11.89 to −2.94; *P* = 0.001), although low-quality trials had no effect (*P* = 0.45). The test for subgroup differences in hospital LOS showed a significant difference between both subgroups (*P* = 0.001). Finally, there were no differences between high- and low-quality trials with regard to ICU LOS (*P* = 0.86).

## Discussion

Systemic inflammation and immune dysfunction are two key features in critical illness. In this context, it may be that provision of parenteral FO-containing LEs in ICU patients receiving PN or EN (pharmaconutrient strategy) represents a promising and attractive therapeutic option. We have updated our previous systematic review by evaluating the effects of FO-containing LEs on relevant clinical outcomes in ten eligible RCTs [15-18,61-66] involving critically ill patients. The main findings of our meta-analysis are that FO-containing LEs may reduce infections and may be associated with a tendency toward fewer MV days compared with SO-based strategies or administration of other alternative LEs in ICU patients. Nonetheless, contrary to our previous findings [10], we did not find a tendency toward reduced mortality, although in EN-based trials FO-containing LEs as pharmaconutrition may be associated with a tendency toward reduced mortality. The benefit in terms of improvement in recovery times, as indicated by the tendency toward a reduction in MV days and hospital LOS, may be due to reduced infections, generally or specifically a lower incidence of VAP, which is a frequent cause of infection in mechanically ventilated patients and is associated with significant attributable morbidity and mortality [67]. The presently reported results notwithstanding, these outcomes should be explored in further well-conducted and adequately powered RCTs.

So far, our systematic review and meta-analysis is the most updated evaluation of the overall effects of FO-based PN strategies in the critically ill. In addition, it is pertinent for intensive care as, according to our inclusion criteria, it contains only RCTs evaluating clinical outcomes in ICU patients. Unfortunately, with the exception of two trials [63,65], most trials included in this systematic review were relatively small studies with fewer than 100 patients and therefore were inadequate to detect a clinically important treatment effect of FO-containing LEs on mortality. However, the advantage of meta-analytic techniques is that they can combine data across studies to discern a more precise treatment effect. In addition, given the wide variety of clinical diagnoses and the heterogeneous population of ICU patients included in this systematic review (sepsis, severe sepsis and/or septic shock, trauma, cancer, pancreatitis, and systemic inflammatory response syndrome), the results and conclusions may be applied to a broad and heterogeneous group of ICU patients.

Since 2012, different meta-analyses [8,9,60] on FO-enriched PN have been published. The differences with our present review are largely due to the variations in the studies included in the reviews. In 2012, Pradelli *et al*. [8] statistically aggregated 23 RCTs in elective surgery and critically ill patients, and, similarly to our findings, they demonstrated that FO-based PN strategies were associated with a significant reduction in infections (RR = 0.61; 95% CI, 0.45 to 0.84; *P* = 0.002). Furthermore, Pradelli *et al*. [8] showed a statistically and clinically significant effect on LOS, both in the ICU (WMD = −1.92; 95% CI, −3.27 to −0.58; *P* = 0.005) and in the hospital (WMD = −3.29; 95% CI, −5.13 to −1.45; *P* = 0.0005), although no effect on overall mortality was found. The results regarding infections in the Pradelli *et al*. study [8] are very similar to our findings, although the inclusion criteria were different, as Pradelli *et al*. included ten trials in adult patients undergoing elective major abdominal surgery and not admitted to the ICU (n = 740). Meanwhile, after aggregating nine RCTs on FO-containing LEs in ICU patients receiving PN, Palmer *et al*. [9] were unable to demonstrate any significant effect on infectious complications (RR = 0.78; 95% CI, 0.43 to 1.41; *P* = 0.41) or on mortality and ICU LOS. Palmer *et al*. [9] included studies published by Wang *et al*. in 2008 [33] and 2009 [61]. However, we excluded the 2008 Wang *et al*. trial [33] because we included a later version of the study that included more patients [61]. Additionally, we excluded two unpublished trials, one by Leiderman *et al*. [54] and one by Ignatenko *et al*. [55]. Both of these trials [54,55] were included in the prior meta-analyses, but they are published only as abstracts, and we were not able to obtain data from the investigators necessary to have these trials included in our review.

In 2014, Chen *et al*. [60] summarized 12 RCTs that administered ω-3 PUFAs through enteral or parenteral routes in ICU patients and showed a tendency toward a reduction in mortality (RR = 0.82, 95% CI, 0.62 to 1.09; *P* = 0.18), although the effect on other clinical outcomes was not analyzed [60].

Is it plausible that FO-containing LEs reduce infections? According to current knowledge, ω-3 PUFAs have different effects on the function and gene expression of the immune cells, affecting cellular and humoral immunity [2-4,6], which may explain their effects on infections in the critically ill. DHA generates protectins, D-series resolvins and maresins, whereas EPA generates E-series resolvins. The immunomodulatory effects of EPA and DHA, in contrast to the ω-6 PUFAs, have been largely recognized for the ability of ω-3 PUFAs to modify leukocyte activity, decrease cell membrane fluidity, alter the production of bioactive mediators and cell signaling, and modulate systemic inflammation by inhibition of cytokine release [2,68]. Intravenous infusion of FO-containing LEs rapidly leads to an incorporation of ω-3 PUFAs in leukocyte cell membrane phospholipids, leading to a reduced production of proinflammatory cytokines because of a higher ratio of ω-3 to ω-6 PUFAs [2,6].

In 2008, Liang *et al*. [32] supplemented patients undergoing radical colorectal cancer resection with FO-enriched PN and showed a reduction in interleukin (IL)-6, a high CD4^+^/CD8^+^ ratio, and higher CD3^+^ and CD4^+^ lymphocytes. These findings suggest that supplementation of EPA and DHA by the parenteral route may support immunocompetent cells in seriously ill surgical patients. Meanwhile, Mayer *et al*. [52] demonstrated a significant improvement in neutrophil function in patients receiving FO-containing LEs, including leukotriene generation and respiratory burst. More recently, in a rat model of sepsis, Terashima *et al*. [69] demonstrated that FO-enriched PN may regulate neutrophil functions, restoring delayed apoptosis, which was associated with an increase in leukotriene B5 (LTB5) production from peritoneal neutrophils. Hecker *et al*. [70], in a very elegant murine model of acute respiratory distress syndrome, demonstrated, among other findings, that parenteral FO decreased leukocyte invasion, protein leakage, myeloperoxidase activity, and cytokine production in the alveolar space. Therefore, they have speculated that ω-3 PUFAs could be beneficial in reducing pulmonary inflammatory complications such as VAP [70]. All of these experimental findings may explain, at least in part, the anti-inflammatory and immunomodulatory effects of ω-3 PUFAs seen in clinical practice.

In sepsis, parenteral FO-containing LEs can result in favorable changes to inflammation and immunity by minimizing inflammation and maximizing resolution of inflammatory response and thus improving patient outcomes. However, resolvin D1 produced from DHA has several actions that stop polymorphonuclear neutrophil infiltration and inhibit microglial cells from expressing inflammatory cytokines in *in vitro* animal models. In addition, resolvin E1 decreases leukocyte infiltration induced by tumor necrosis factor (TNF)-α [71]. Therefore, oversuppression may potentially lead to immunosuppression, although there are conflicting data on the effect on cytokine expression and immunological markers, as no documented reports of negative hemostatic outcomes from FO-containing LEs in sepsis/severe sepsis have been published.

The investigators in the clinical trials included in our systematic review evaluated mechanistic effects of FO-containing LEs. Hall *et al*. [17] demonstrated a significant reduction in C-reactive protein (CRP) mean values in the FO-enriched PN group, although Burkhart *et al*. [18] were unable to find differences in IL-6, IL-8, IL-10 and CRP levels between patients supplemented and non-supplemented with FO. Meanwhile, after comparing two alternative LEs, Gultekin *et al*. [66] found a significant reduction in LTB4 and CRP levels in FO-supplemented patients, whereas IL-6 and TNF-α levels were not different between groups. Previously, Barbosa *et al.* [64] demonstrated a significant reduction in plasma IL-6 and IL-10 levels in the FO group. Similarly, Wang *et al*. [61] showed an increase in IL-10 levels and human leukocyte antigen DR expression, as well as a concomitantly significant reduction in CRP levels, in patients with severe acute pancreatitis.

The strength of our meta-analysis is based on the fact that, as in previous meta-analyses, we used several methods to reduce bias (comprehensive literature search, duplicate data abstraction, specific criteria for searching and analysis) and focused on clinically important primary outcomes in ICU patients. Nevertheless, we are aware that our meta-analysis has several limitations, such as the limited number of trials included to evaluate different outcomes. In addition, the effect on infections is driven mostly by the large RCT by Grau-Carmona *et al.* (N = 159) [65]. This trial explains 52% of the signal and is thus an unstable estimate, which increases slightly after the sensitivity analysis excluding the Hall *et al*. study [17] (RR = 0.64; 95% CI, 0.44 to 0.94; *P* = 0.02; heterogeneity *I*^2^ = 0%). In addition, most of data about MV days and ICU and hospital LOS were extracted from the original reports, which may underestimate the average duration in those cases where LOS of non-survivors was included in the calculation. Finally, there are two unpublished abstracts [54,55] from which we could not obtain sufficient data to include in our meta-analysis. These factors may limit the reliability of our estimates and weaken the strength of our conclusions.

## Conclusions

In this updated systematic review and meta-analysis, we demonstrate that parenteral FO-containing LEs in critically ill patients may be able to significantly reduce the incidence of infectious complications and also could be associated with a reduction in the duration of MV and hospital LOS. Our results are generalizable to patients receiving some form of artificial nutrition in the ICU setting. Nevertheless, according to the current literature, there is inadequate evidence to give a strong recommendation on the routine use of FO-containing LEs in PN and/or as a pharmaconutrient strategy in enterally fed critically ill patients. Additional large-scale and well-designed RCTs, which should be aimed at confirming our observations, are required and warranted.

## Key messages

- Intravenous FO-containing LEs are rich in ω-3 PUFAs, which exhibit anti-inflammatory and immunomodulatory effects.
- These FO-containing strategies may be able to significantly reduce the incidence of infectious complications and also could be associated with a reduction in the duration of MV and hospital LOS in critically ill patients.
- FO-containing LEs as pharmaconutrient strategies in enterally fed patients may be able to reduce mortality.
- So far, there is inadequate evidence to give a final recommendation on the use of FO-containing LEs as a ω-6 fatty acid–reducing strategy in ICU patients who require PN and/or as a pharmaconutrient strategy in enterally or orally fed patients.

## Abbreviations

ARDS: Acute respiratory distress syndrome
CI: Confidence interval
C.Random: Concealed randomization
CRP: C-reactive protein
DHA: Docosahexaenoic acid
EN: Enteral nutrition
EPA: Eicosapentaenoic acid
FO: Fish oil
ICU: Intensive care unit
IL: Interleukin
ITT: Intention to treat
LCT: Long-chain triglyceride
LE: Lipid emulsion
LOS: Length of stay
LTB4: Leukotriene B4
LTB5: Leukotriene B5
MCT: Medium-chain triglyceride
MV: Mechanical ventilation
NA: Non-attributable
NR: Non-reported
OO: Olive oil
PN: Parenteral nutrition
PO: Oral nutrition
PUFA: Polyunsaturated fatty acid
RCT: Randomized controlled trial
RR: Risk ratio
SIRS: Systemic inflammatory response syndrome
SO: Soybean oil
TNF: Tumor necrosis factor
TPN: Total parenteral nutrition
VAP: Ventilator-associated pneumonia
WMD: Weighted mean difference
